# Microalgae in Microwell Arrays Exhibit Differences with Those in Flasks: Evidence from Growth Rate, Cellular Carotenoid, and Oxygen Production

**DOI:** 10.3389/fpls.2017.02251

**Published:** 2018-01-10

**Authors:** Ping Zhang, Yan Xiao, Zhe Li, Jinsong Guo, Lunhui Lu

**Affiliations:** ^1^Department of Environmental Science and Engineering, College of Urban Construction and Environmental Engineering, Chongqing University, Chongqing, China; ^2^CAS Key Lab of Reservoir Environment, Chongqing Institute of Green and Intelligent Technology, Chinese Academy of Sciences, Chongqing, China

**Keywords:** microwell arrays, physiological response, *Chlorella vulgaris*, Raman spectroscopy, non-invasive microtest

## Abstract

Microalgae are cultivated in macro-scale reactors traditionally and the relevant knowledge is based on bulk analysis. Whether the knowledge and laws are true for cells under micro-cultivation is still unknown. To better understand microalgal physiology, micro-cultivation of microalgae, and unicellular tracking and analysis of its response *in vivo* is necessary. In the study, cellular responses of *Chlorella vulgaris* to micro-cultivation is studied, with cells in flasks as a control. Five different microwell depths ranging from 10 to 200 μm with a fixed diameter of 100 μm, and four diameter levels from 30 to 200 μm with a fixed depth 60 μm were investigated. Unicellular dynamics showed that cell number differences among various types of microwells with different initial cell numbers decreased as cultivation processed. Besides, the specific growth rate of *C. vulgaris* on microwell arrays was much higher than that in flasks and so cells on microwell arrsys can be much sensitive to pollutants. Thus, the interesting characteristics may be used in cell sensor applications to enhance sensitivity. The specific growth rate of *C. vulgaris* on microwell arrays decreased gradually as the microwell diameter increased from 30 to 200 μm while presented a unimodal trend as depth decreased from 200 to 10 μm. Furthermore, we used Raman Spectroscopy and Non-invasive Micro-test Technique to analyze cellular responses in microwells for the first time to track the changes *in vivo*. Results indicated that unicellular carotenoid content increased as microwells became larger and shallower. The flow rate of oxygen rose gradually as the depth increased from 10 to 100 μm, but then decreased rapidly as the depth deepened to 200 μm. In fact, it is a combined result of cell physiology and density. In summary, cells in microwells with the diameter/depth ratio ~1 owned the highest specific growth rates and oxygen flow rates. Simulations also suggested that better mass transfer occurred in microwells with higher diameter-to-depth ratios.

## Introduction

*Chlorella vulgaris* (*C. vulgaris*) is a unicellular green microalga that commonly exists in fresh water environments. Due to the fast-growing nature and the capability of bioactive substance production (Cardozo et al., 2007; Singh et al., 2011), it has been widely used as an important economic algal species in bio-engineering industry. In addition to its bulk cultivation in various large-scale photobioreactors, a recent application of this tiny creature is to employ them as cell sensors for the detection of heavy metals or herbicides (Chouteau et al., 2005; Shing et al., 2013). This “scaled down approach” requires a limited number of cells to sensitively and accurately respond to physical or chemical signals from the culture environment within a certain time range and spatial boundary. However, the detection limit of cell sensors is often not low enough for some of the targeted substances (Shing et al., 2013). It varies according to the bioreceptor used (Giardi et al., 2001). A group of cells can have good resistance toward external disturbances and shocks thus may be less sensitive to toxicant (Chouteau et al., 2004). Studies by Chouteau et al. (2004) indicated that conductometric biosensors using algae was more sensitive than bioassays to detect low levels of cadmium ions. So the applicability of a small number of cells or even a single cell as bioreceptor may probably raise the sensitivity of detection. However, most knowledge at present on the cellular physiology of *C. vulgaris* is from traditional cell culture methods, which only allow bulk cultivation and analysis of cellular responses. Therefore, further understanding of the physiology of *C. vulgaris* at very small populations or even single-cell level is important for improved applications (Rowat et al., 2009; Lu et al., 2013; Osada et al., 2014).

Unicellular responses are often obtained using the microwell array cell culture system, as it can hold cells at specific positions, making the cell behavior tracking process easier (Ochsner et al., 2007; Hwang et al., 2009; Park et al., 2011; Espulgar et al., 2015). Microwell array is a type of microelectromechanical system that is composed of multiple micropore arrays with pore sizes at micro-scales, like miniaturized well plates. The microwell array cell culture system was first introduced in the field of tissue engineering for cell culture (Taylor and Walt, 2000; Rettig and Folch, 2005; Charnley et al., 2009) and for studying cell matrix interactions (Loessner et al., 2013), unicellular physiology (Inoue et al., 2001) and so on mainly because it can mimic the cellular physiological 3D environment. Later, microwell arrays were increasingly put into use in the aspect of chemistry and plant cell cultivation (Zheng et al., 2013). Inoue et al. studied the division characteristics of *Escherichia coli* at single-cell level using a micro-chamber array system (Inoue et al., 2001), and the real-time cellular responses of *Saccharomyces cerevisiae* to various concentrations (1 nM−100 mM) of mating pheromone at single-cell resolution were monitored by Park et al. (2006) through applying a microwell array. Both studies were based on a time-lapse microscope to monitor unicellular behavior. Furthermore, a microfluidic photobioreactor array system was used to study the optimal light intensity for oil production by *Botryococcus braunii* (Kim et al., 2014). In this research, 64 different light conditions were generated by applying a high-throughput microfluidic microalgal photobioreactor array. However, in these assays, the detecting methods of the cellular responses in microwells were limited to microscopic methods or microscopy combined with fluorescence staining (Yamamura et al., 2005; Kim et al., 2014), along with electrochemical methodology (Sardesai et al., 2011). Though intuitive, these methods have limited access to further information such as structural information of substance inside a certain cell. Microscopic methods can only obtain cell density and cursory morphology information. The combination of fluorescence staining could yield more information regarding internal substances, while this is just limited to those which can be detected with fluorescence (Shing et al., 2013). Due to the limited cellular endpoint obtaining methods, unicellular studies for *C. vulgaris* have scarcely been explored.

The Micro-Raman spectroscopic technique (RS) and the Non-invasive Micro-test Technique (NMT) are both non-invasive techniques capable of probing into a single cell. RS is a fast and sensitive analytical method that elucidates the structural information of the molecules (Barletta et al., 2015). RS applies a microscope objective to focus the laser onto a specific sample and thus, is usually used to probe single cellular compositions (Huang et al., 2010). NMT is normally used to obtain molecular or ionic concentration and flux information of a small number of fixed cells or tissues (Zhang et al., 2009; Wan et al., 2011). Thus, RS and NMT techniques can help to track cellular compositional and physiological states easily.

To better understand microalgal physiology and explore the differences of cell physiology between microalgae cultivated in micro-scale and traditional macro-scale, we hypothesized that cells under micro-cultivation conditions can respond differently from that cultivated traditionally. Besides, we also hypothesized microwells with different dimensions not only have different confining capabilities—the ability to keep cells from running away from microwells—but also different mass-exchanging capacities, thus interfering with cellular responses. In the present study, we designed microwell arrays with different dimensionalities and studied the relevant cellular responses of *C. vulgaris* on these microwell arrays using non-invasive techniques with a macro-scale culture as a control. We believe that the newly applied RS and NMT non-invasive techniques will provide new information on cellular response of *C. vulgaris* at single-cell level and constrained small populations and will be helpful for further design of *C. vulgaris* cell sensors and other applications.

## Materials and methods

### Design and photolithographic fabrication of microwell arrays

**Figure 1:**
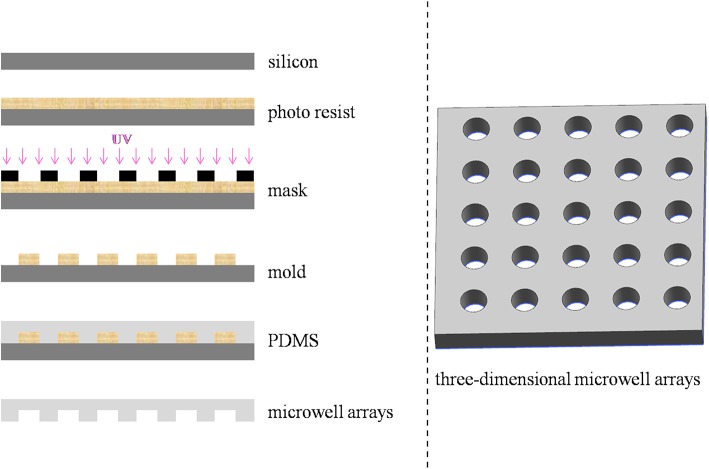
Schematics of the fabrication procedure of the microwell arrays.

Different microwell arrays were designed and fabricated. Specifically, microwell arrays with triangular, square, hexagonal, and cylindrical well shapes (a fixed depth and diameter of 60 and 100 μm, respectively) as well as microwell arrays with four different diameters ranging from 30 to 200 μm with a fixed depth of 60 μm and five depths ranging from 10 to 200 μm with a diameter of 100 μm were designed. The process of lithography for the microwell arrays was shown in Figure 1. Photolithography was performed at the Wenhao Chip Technology Company of Suzhou, China. The fabrication processes and methods were adapted from studies by Rettig and Folch (2005). In brief, silicon wafers were first baked to improve photoresist adhesion. Then, SU-8 photoresist with the specific models SU8-2010, SU8-2025, SU8-2035, SU8-2075, and SU8-2150 for microwell arrays with depths 10, 30, 60, 100, and 200 μm (MicroChem Corp, Newton, MA) were spread immediately at ~500 rpm for 10 s, and spun for 30 s at 3,500, 3,000, 2,000, and 2,800 rpm to yield feature heights of H = 10, 30, 60, 100, and 200 μm, respectively. Immediately after spinning, wafers were solidified on a hot plate with a programmed temperature control function. The silicon wafers were covered with mask and then exposed to UV light. They were then etched using a standard reactive ion etching process. After removing the photoresist from the etched wafer, a 15:1 v/v mixture of polydimethylsiloxane (PDMS) polymer and cross-linker (Silpot 184W/C, Dow Corning Toray, Tokyo, Japan) was poured onto the cast wafer, which were degassed under vacuum and cured at 90°C for 2 h, and the resulting cured PDMS sheets with different microwell patterns were peeled from the mold.

### Mass transfer in microwell arrays

Three-dimensional microwell arrays are commonly used for cell culture applications. During the cultivation procedure, mass transfer of O_2_ or nutrition diffusion is of significant importance to enhanced cell viability (Randall et al., 2011). To achieve a better quantitative understanding of the diffusion characteristics in the microwells, we simulated the mass transfer of CO_2_ in different microwell dimensions. We simulated cylindrical microwells of diameters 200, 100, 50, and 30 μm with a fixed depth of 60 μm and microwells of depths 200, 100, 60, 30, and 10 μm with a fixed diameter of 100 μm. The individual 3D microwell arrays were placed in the bottom of a petri dish with culture medium. Stationary solutions of the spatial variation of CO_2_ concentration were obtained by solving the diffusion equation:

(1) $$\frac{\partial \text{c}}{\partial t}+\nabla -D\nabla \text{c}=0$$

Here, c is the CO_2_ concentration, and *D* is the diffusion coefficient of CO_2_ in the medium. For the boundary conditions, we assumed the CO_2_ concentration at the medium-air interface to be constant and equal to 0.03 mM. Simulations were carried out using MATLAB R2014a.

**Figure 2:**
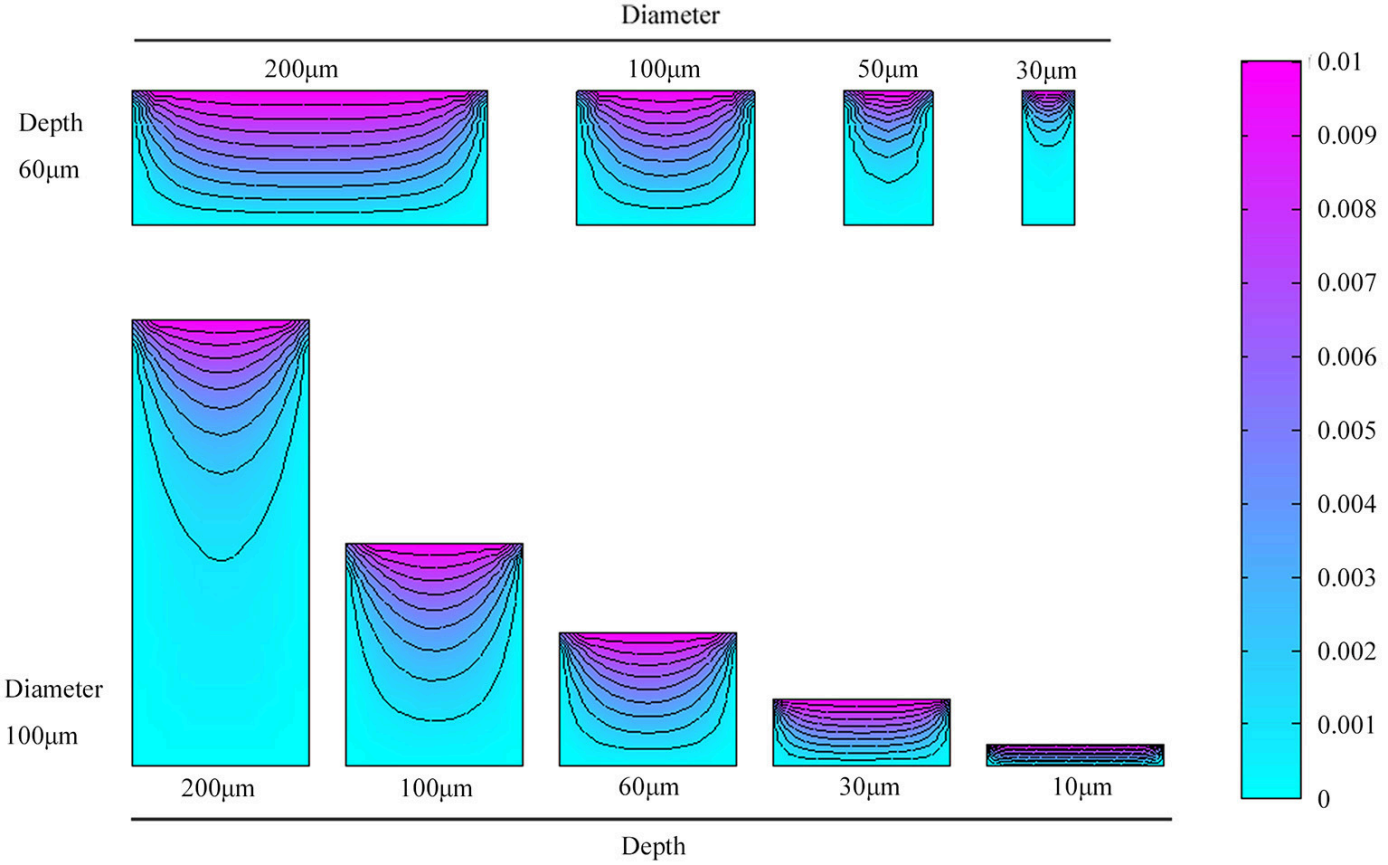
Numerical simulations and comparison of mass transfer in microwells of different diameters and depths.

Numerical simulations of mass transfer in various microwell dimensions are presented in Figure 2. The first and second rows show mass transfer in microwells with different diameters and depths, respectively. As microwells became narrower and deeper, larger volume fractions of carbon-deficient space existed. In conclusion, better mass transfer occurred in microwells with higher diameter to depth ratios.

### Algae and pre-cultivation conditions

The microalgae used in the experiment was the green alga *Chlorella vulgaris* (FACHB-32) obtained from the Freshwater Algae Culture Collection of the Institute of Hydrobiology, Chinese Academy of Sciences. It was precultivated according to the method described in our previous article before seeding (Zhang et al., 2017). Simply, algae was precultivated in BG11 medium with an aeration rate of 70 mL/min in an Erlenmeyer flask with the volume of 1,000 mL. Samples were cultivated under 25 ± 2°C, with a 12:12 h light/dark cycle at a light intensity of 43 μmol·m^−2^·s^−1^, illuminated from all around by cool-white fluorescent lamps.

### Seeding and statistics of *C. vulgaris* cells on PDMS microwell arrays

Algal samples were diluted to a cell density of 1 × 10^5^ cells/mL before seeding. Microwell arrays obtained from section Designation and Photolithographic Fabrication of Microwell Arrays were pretreated with high-speed double-distilled water to expel air in the wells and were then placed in a glass petri dish with 50 mL of algal samples. Cells were sorted randomly in an independent fashion into individual microwells by gravity so that the number of cells per well followed a Poisson distribution on each microwell array. Similar results were also discussed by Ino et al. (2008). The whole seeding process took 1 h. After seeding, microwell arrays were carefully taken out with tweezers, washed carefully in sterile BG11 medium and then placed the PDMS microwell array chip in a confocal petri dish with 4 mL of sterile BG11 medium. Pictures were taken at 10 × magnification on an inverted Olympus microscope (Olympus IX73, Japan). Microwells with cells were counted respectively, and statistical assessments were made on different microwell arrays. After statistical calculations, microwell arrays were cultivated under 25 ± 2°C at a light intensity of 40 μmol·m^−2^·s^−1^ with a 12:12 h light:dark cycle in a confocal petri dish for a period of 9 days. The algae with a cell density of 1 × 10^5^ cells/mL cultured in Erlenmeyer flask with a volume of 250 mL under the same condition as that introduced to microwell arrays was used as a control. It was manually shook three times a day to allow mixing. Ten replicates were carried out for both microwell arrays and flasks in the experiment.

### Specific growth rate for *C. vulgaris*

Cell numbers in each microwell were counted every other day under a microscope (Olympus IX73, Japan) with a magnification of 40 × to track cell density changes. Specific growth rate was measured in the exponential growth phase, according to Guillard (1973): μ = *ln (x*_2_*/x*_1_*)/(t*_2_*-t*_1_*)*, where *x*_2_ and *x*_1_ are the cell densities at sampling days of *t*_2_ and *t*_1_, respectively.

### Carotenoid determination using the raman spectroscopic method

A Raman spectroscopic technique was used to determine the relative cellular carotenoid content at the end of the experiment—on the 9th day. Detailed methods can be found in the study by Zhang et al. (2017). In brief, a Confocal Raman Microscope (inVia-Reflex made by Renishaw England) with a 532 nm laser device was used in the experiment. The laser was focused onto the sample with a 50 × /0.6NA Nikon objective. All spectra were collected in the extended mode with a resolution ≤1 cm^−1^. Microalgal cells were subjected to Raman conditions with an integration time of 10 s, a laser power of 0.1 mW and a 1 × accumulation times to obtain unicellular carotenoid content on different microwell arrays. A total of 20 algal cells were selected randomly to collect Raman information and to obtain unicellular relative carotenoid content information in a specific microwell array with different dimensionalities and in the control.

### Oxygen flow rate determination of algal cells by NMT

Oxygen flow rates showed the efflux and influx information of oxygen in which positive results represented efflux of oxygen and vice versa. The oxygen flow rates of microalgal cells were determined under an inverted microscope using a microelectrode with a diameter of ~20 μm on a non-invasive micro-test system (NMT-100 series System, Younger USA LLC, Amherst, MA). Cells in microwells were regarded as a whole in the detecting process. Ten microalgal clusters of similar density and size were detected and averaged to obtain the mean flow rate of oxygen for a specific sample.

### Data analysis

All figures and analysis of variance (ANOVA) were created and done by the data processing software OriginPro 8.0. The Raman intensities of characteristic peaks were directly acquired from the Raman software WIRE 3.4, and the peak at 1,523 cm^−1^ was selected to quantify carotenoids in a single cell (Cannizzaro et al., 2003). For a specific sample, the peak intensities at 1,523 cm^−1^ were averaged for 20 algal cells. The oxygen flow rate of microalgal cells for a specific sample was an average of 10 algal clusters. Data in this study are presented as the means ± standard deviations.

## Results

### Cell distributions on different microwell arrays

**Figure 3:**
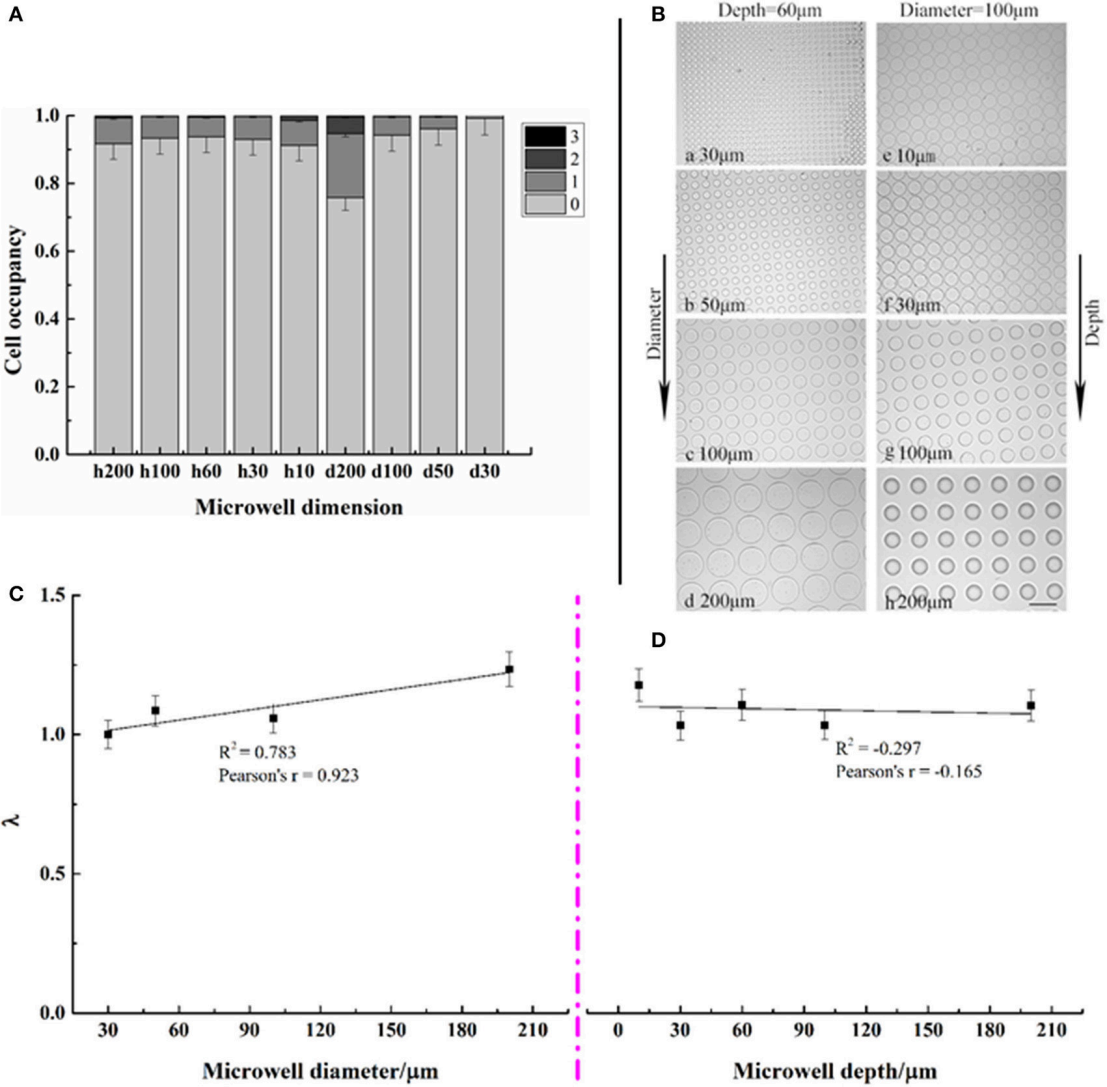
Distribution of microwell occupancies for different microwell dimensions **(A,B)** as well as correlation analysis between the average number of cells per well λ and microwell dimensions **(C,D)**. Error bars in the figures show the standard deviation. (*n* = 10).

Cell distributions—the number of cells per well—of *C. vulgaris* on various dimensions of microwell arrays are shown in Figures 3A,B. Results indicated that most microwells were empty with the seeding cell density 1 × 10^5^ cells/mL and settling time of 1 h. No wells with more than three cells were found for all types of microwell arrays. Occupancy of microwells containing a single cell, i.e., single-cell occupancy, did not change significantly (*p* > 0.05) as the well depth increased from 10 to 200 μm. However, it decreased rapidly as well diameters decreased. When the well diameter was 200 μm, single-cell occupancy was 5.65%; nevertheless, as the well diameter decreased to 30 μm, only 0.4% of wells contained a single cell, indicating that single-cell occupancy for different types of microwell arrays was closely related to well diameter while had little relationship with well depth. Figure 3B showed microwell arrays with various dimensions after seeding, taken at 10 × magnification on an inverted Olympus microscope.

The number of cells per well followed a Poisson distribution on each microwell array. The values of λ, i.e., the averaged cell numbers per well for each type of microwell array, were correlated with the depths and diameters in Figures 3C,D, respectively. It presented a positive correlation with well diameters, with coefficients of ~0.8. However, little correlation with well depth existed.

### Cell density and specific growth rates for *C. vulgaris* in microwell arrays and flasks

**Figure 4:**
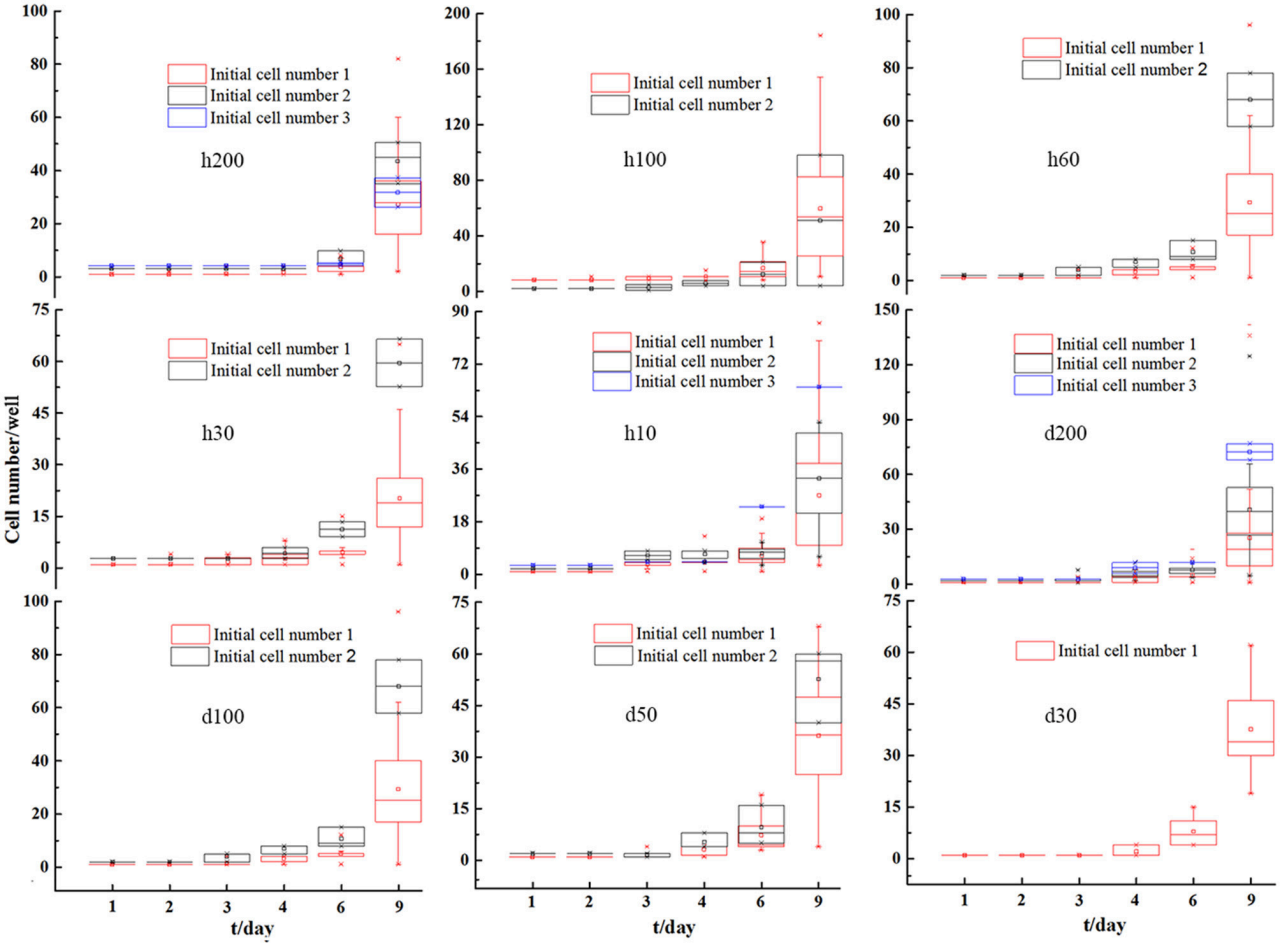
Averaged single microwell cell density changes for *C. vulgaris* with initial cell number 1, 2, and 3 on microwell arrays with different depths and diameters. Error bars in the figure show the standard deviations. (*n* = 10).

*C. vulgaris* cells were counted every other day to track cell density changes over time. Cell density changes in microwells with initial cell numbers of 1, 2, and 3 were averaged respectively, and relevant results are presented in Figure 4. Overall, it presented a gradual increasing trend over time for all microwell dimensions, and the differences for cell densities among various types of microwells with different initial cell numbers were reduced over time. These results indicated that smaller differences in heterogeneity among cells existed as cell division proceeded on a population scale.

**Figure 5:**
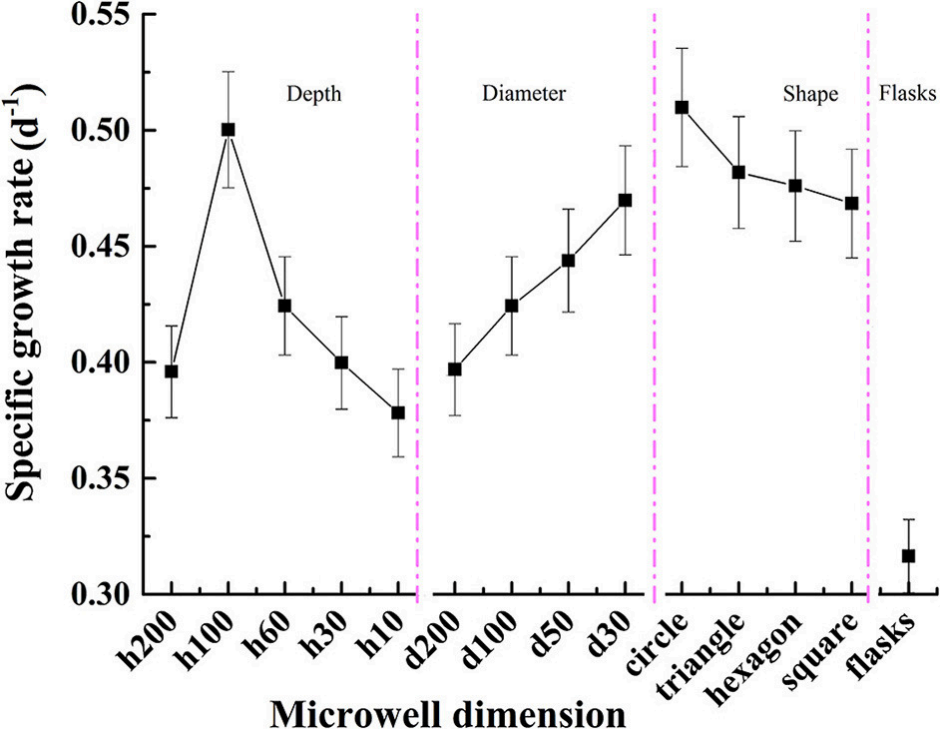
Specific growth rate changes for *C. vulgaris* cells on different microwell arrays and in flasks. Error bars in the figure show the standard deviations (*n* = 10).

The specific growth rates for *C. vulgaris* on different microwell arrays and in flasks are presented in Figure 5. Cells on microwell arrays showed much higher specific growth rates than those cultivated in flasks. The highest specific growth rate for *C. vulgaris* on microwell arrays was ~61.2% higher than that of cells cultivated in flasks. The cellular specific growth rate rose gradually as microwell diameter decreased; it increased from 0.397 in microwell arrays with a diameter of 200 μm to 0.470 as well diameter decreased to 30 μm, which was ~18.4% higher. Unlike the trend with diameters, the specific growth rates for *C. vulgaris* on microwell arrays with different depth showed a unimodal tendency as microwell depth rose from 10 to 200 μm. The highest specific growth rate for *C. vulgaris* on microwell arrays were measured on microwell arrays with well depth of 100 μm. There were no significant differences among specific growth rates on microwell arrays with triangular, square and hexagonal well shapes. Meanwhile, the growth rate was slightly higher in cylindrical microwell arrays.

### Unicellular carotenoid content for *C. vulgaris* by the raman spectroscopic method

**Figure 6:**
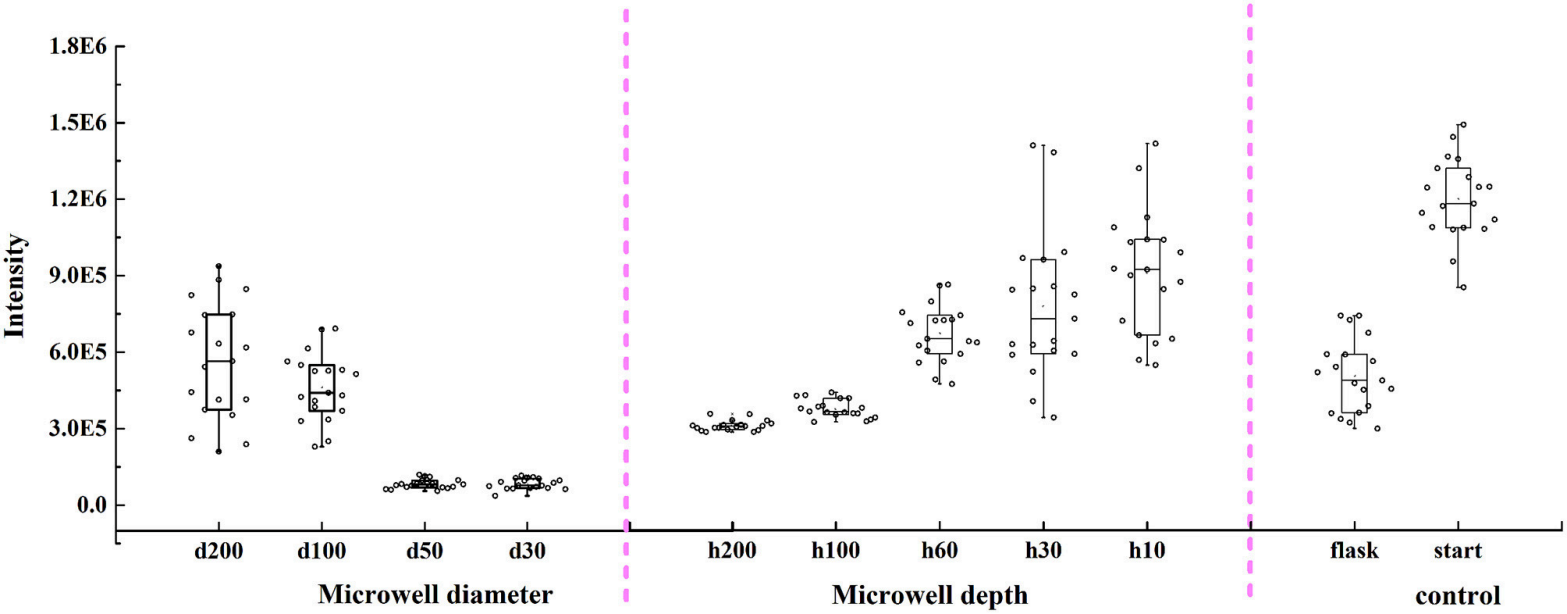
Changes of unicellular carotenoid content in microwell arrays with different diameters, depths, and flasks by Raman spectroscopic method. Error bars in the figure show the standard deviations (*n* = 20).

Changes for unicellular carotenoid content of *C. vulgaris* in different types of microwells and in flasks are shown in Figure 6. Results indicated that unicellular carotenoid content of *C. vulgaris* in different types of microwells was lower than that at the start of the experiment, where microalgal cells were obtained right after enrichment by aerating. Additionally, it presented a monotone rising trend as microwell diameters increased from 30 to 200 μm and microwell depths decreased from 200 to 10 μm. There were no significant differences (*p* > 0.05) among unicellular carotenoid content in flasks, microwells with diameters of 100 and 200 μm (depth 60 μm), and depths of 60 and 30 μm (diameter 100 μm). However, it was relatively higher for cells in microwells with a depth of 10 μm. The unicellular carotenoid content distribution also suggested that cellular heterogeneity existed both in microwells and flasks.

### Oxygen flow rate changes for *C. vulgaris* by NMT

**Figure 7:**
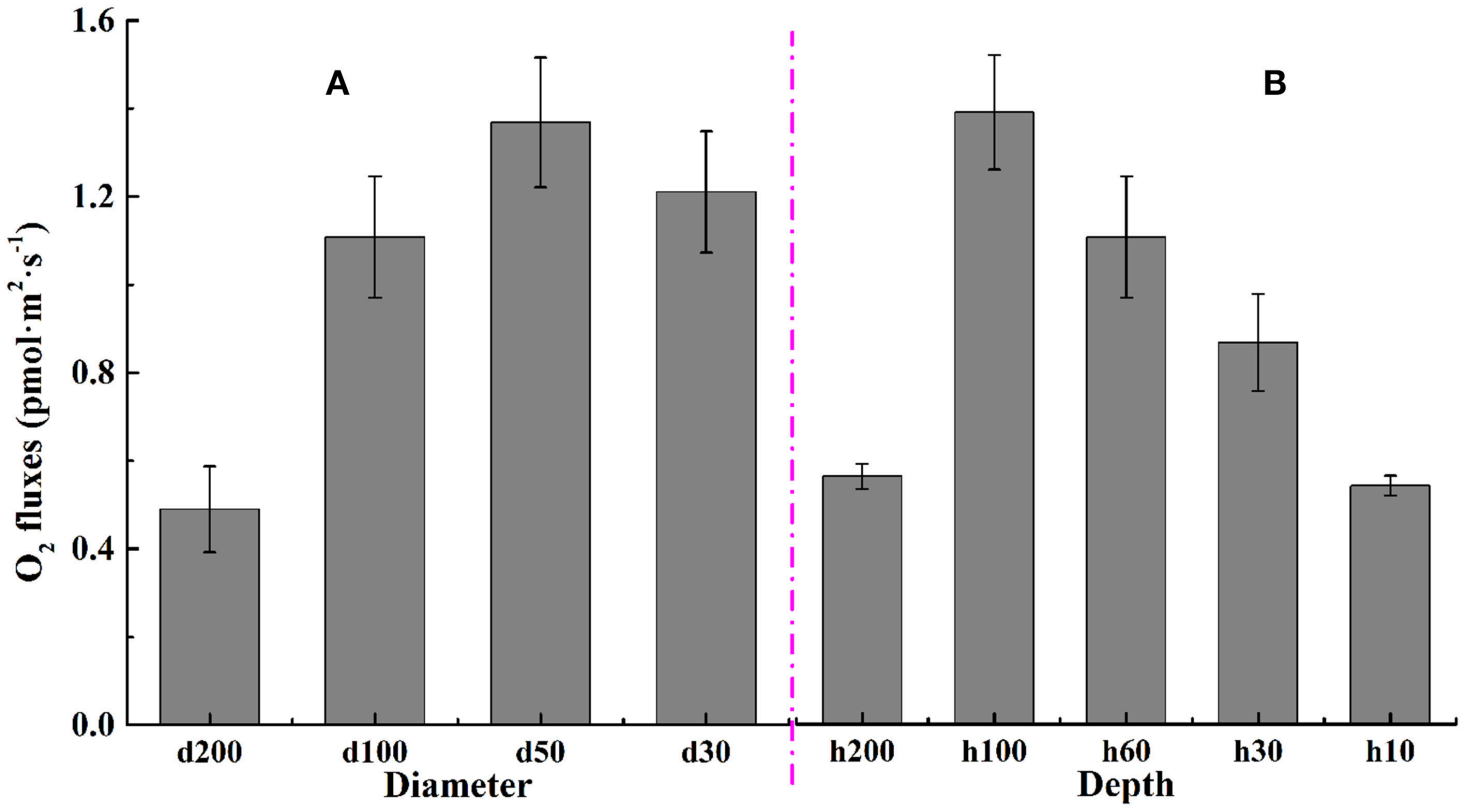
Oxygen flow rate for *C. vulgaris* in microwell arrays of different **(A)** diameters and **(B)** depths. Error bars in the figure show the standard deviations. (*n* = 10).

Oxygen flow rates of *C. vulgaris* in microwells were obtained using the Non-invasive Micro-test Technique, and the relevant flow rate results for cells in different types of microwells are displayed in Figure 7. Results in Figure 7A showed that there were no significant differences (*p* > 0.05) for oxygen flow rates of microalgal cells in wells with diameters of 100 and 30 μm. The maximum oxygen flow rate was detected in wells with a diameter of 50 μm where the diameter/depth ratio was nearly 1. This finding was in good agreement with the specific growth rate results, where the maximum was also obtained on the microwell arrays with the diameter/depth ratio ~1.

From results presented in Figure 7B, there existed an obviously unimodal changing tendency between oxygen flow rates and well depth. It rose gradually as microwell depth deepened, with the maximum obtained at the well depth of 100 μm, where the diameter/depth ratio was right 1. The flow rate then decreased as the depth increased more to 200 μm.

## Discussions

Eco-physiological responses of *C. vulgaris*, e.g., changes in cellular elemental and bio-macromolecular compositions have been widely studied and evaluated, based on an average approach to the *C. vulgaris* population in experimental systems. This approach was scientifically reasonable and technologically feasible, as most of the physical and chemical analytical methods require abundant algal samples. However, this limited the acquisition of further cell response information at single-cell level.

In the present study, we designed different microwell dimensions to study *C. vulgaris* responses under micro-cultivation conditions and various diffusion gradients. Changes for unicellular carotenoid content in these different microwells indicated that cells in microwells with higher diameter/depth ratios possessed higher carotenoid content. Besides, it presented a positive correlation between unicellular carotenoid content and diameter/depth ratio, which was inconsistent with cell density and specific growth rate changes, as they were related not only to mass transfer but also the confining capability. Probably because microwells with higher diameter/depth ratios have better mass transfer conditions, as could be clearly identified from the mass transfer simulation in section Mass Transfer in Microwell Arrays. Similar results were also discussed by Randall et al. (2011), who highlighted the importance of oxygen diffusion in three-dimensional microwell array cell culture systems to enhance cell viability. Besides, cells cultivated in flasks without aeration possessed much lower unicellular carotenoid content than those with bubbling, even lower than that in microwells with well depth 10, 30, and 60 μm. This may possibly due to the better mass transfer process and CO_2_ supply. It seems that cells with better physiological states have higher unicellular carotenoid content. Though some stressed conditions can facilitate the accumulation of carotenoid such as nitrogen or high light stressed conditions (Lamers et al., 2010), some studies also demonstrated the fact that relatively higher CO_2_ can facilitate the accumulation of caroteinoid (Reddy et al., 2014). Besides, the deficiency of CO_2_ can lead to lower photosynthesis efficiency and carotenoid content (Gilles et al., 2008; Singh and Singh, 2014).

Compared with well diameter, depth had a greater impact on specific growth rates for *C. vulgaris* cells. The specific growth rate presented a unimodal trend toward depth as microwell depth increased from 10 to 200 μm. The trend was likely caused by the combination of mass transfer and cell dislodgement processes. When the well is deeper than the critical depth, the transfer of carbon dioxide, and other nutrients to the cells becomes difficult, leading to poor cellular growth and reproduction capacity (Randall et al., 2011); conversely, cells were inclined to escape from the microwells if the well was too shallow. Additionally, the results indicated that the specific growth rates for cells on microwell arrays were much higher than the growth rate of cells cultivated in flasks. It showed the different cellular responses between cells cultivated under micro-cultivation and traditional macro-cultivation conditions. This may be because cells cultivated on microwell arrays have better light and mass transfer conditions. Additionally, there exists very little competition among cells on microwell arrays compared with that in flasks and every cell nearly possesses the same niche on microwell arrays. All these advantages are far beyond that owned by macro-scale cultivations. So the responsive microalgal cultivation method can be used in cell sensor applications to enhance the sensor sensitivity. Future studies would be focused on how to combine microwell arrays or similar cultivation devices with transducers to achieve high-sensitivity.

The oxygen flow rate of *C. vulgaris* cells on microwell arrays is a combined result of cell physiology and cell density. The changing trend for oxygen flow rates at different microwell depths presented a similar unimodal tendency as that of the specific growth rate. The lowest two oxygen flow rates were found in microwells with depths of 10 and 200 μm, likely because algal cells in shallower microwells were inclined to be dislodged from the wells. Because cells in shallower microwells have sparse cell distribution, and vice versa for cells in overly-deep wells. In summary, the highest oxygen flow rate was detected in algal cells from microwells with the diameter/depth ratio around 1, which was consistent with the specific growth rate results.

Cell occupancy was related to microwell dimensions in the case of the consistent seeding cell density and time (Rettig and Folch, 2005). According to the research by Rettig and Folch (2005), the total number of trapped single cells increased as the microwells became deeper and narrower, which did not seem to be the case in our study. In the present study, there were no significant differences among single-cell occupancies on microwell arrays with different depths. Furthermore, as the wells became narrower, single-cell occupancy decreased. Multiple cells tended to occupy wider and shallower wells in our study, likely because of the different sedimentation performances between different cell types and physiological statuses. This observation is also apparent in the results presented in the Supplementary Materials, in which it indicated that *C. vulgaris* cells from batch culture settled more easily onto the microwell arrays than cells from aerating culture. These findings suggested that cell distributions on microwell arrays presented different characteristics in accordance with seeding cell density, settling time, cell type, and status.

Cell occupancy of the microwells was relatively low compared with other researches (Osada et al., 2014). This may possibly due to the planktonic characteristics of *C. vulgaris*. To improve the occupancy, other manual intervention could be employed such as optical tweezers and negative pressure methods (Luo et al., 2007; Osada et al., 2014). However, optical tweezers may have negative effects on cells if too high laser power is used (Rasmussen et al., 2008). Moreover, the device is expensive and hard for realization for most researchers. The usage of negative pressure proposed by Osada et al. (2014) were proved to be highly effective for cell trapping in microcavity arrays. However, this is suitable only for through holes and cannot be applicable in the blind-hole microwell arrays in the case of our study.

In summary, results from the study demonstrated the applicability of our microwell arrays to lowering the detection limit of microalgal biosensors through the following two plausible approaches. For one thing, limited cell numbers in microwell arrays, which could be achieved through the smaller size of microwells, could sensitively respond to the changes of external environment. For the second, the possible detective method, e.g., RS or NMT in the present study, could sensitively measure the physiological response at cell level. Nevertheless, there have been trade-offs between the size of microwell, e.g., the depth/width ratio, and the number of cells cultivated in microwell arrays. Larger microwells could hold more microalgal cells, which could possibly decrease the capability of cell assemblages in microwell to detect external environment. Smaller groups of microalgae in microwells seems more sensitive to detect external environment, but might be limited by the diffusion between external environment and microwells. Future study should be carried out on this issue which we believe will be meaningful to develop microalgal biosensors with relatively high sensitivity and resolution in response to changes in the external environment of the cells.

## Conclusions

This research demonstrated the feasibility of studying cells in a small population or even at single-cell levels. Results indicated that the cellular responses for *C. vulgaris* on microwell arrays were quite different from cells cultivated in bulk scale, i.e., in flasks. The specific growth rate was much higher for cells on microwell arrays than that in flasks. It showed a unimodal trend toward microwell depth, with the maximum in microwells with the diameter/ratio ~1. Similar variation tendencies were detected for oxygen flow rate while unicellular carotenoid content was higher in shallower (fixed diameter 100 μm, depth ranging from 10 to 200 μm) and larger (fixed depth 60 μm, diameter ranging from 30 to 200 μm) microwells—the better cellular physiology, the higher unicellular carotenoid content. Moreover, cellular heterogeneity decreased to some extent with continued cultivation, as could be seen from averaged single microwell cell density changes.

Our study demonstrated that cells under micro-cultivation conditions respond differently from that cultivated traditionally and that microwells with different dimensions not only have different confining capabilities but also different mass-exchanging capacities, thus interfering with cellular responses.

## Author contributions

PZ performed the experiment, collected data, and draft the MS. YX and ZL initiate the idea of the research, analyzed data, and made revision of the MS. JG and LL participated discussion of the results.

### Conflict of interest statement

The authors declare that the research was conducted in the absence of any commercial or financial relationships that could be construed as a potential conflict of interest.
