# Supplementary material for: Microalgae in Microwell Arrays Exhibit Differences with Those in Flasks: Evidence from Growth Rate, Cellular Carotenoid, and Oxygen Production
Source: Front Plant Sci. 2018 Jan 10;8:2251. doi: 10.3389/fpls.2017.02251 (PMC5770892; doi:10.3389/fpls.2017.02251)
Supplement: Supplementary file 1 [file DataSheet1.DOCX]

**Cellular physiological and kinetic responses of *Chlorella vulgaris* to different microwell arrays**

Ping Zhang^a,b^, Yan Xiao^b^, Zhe Li^a,b^, Jinsong Guo^a^, Lunhui Lu^b^

^a^College of Urban Construction and Environmental Engineering, Chongqing University No. 174, Shazheng Street, Shapingba, Chongqing, 400045, China.

^b^CAS Key Lab of Reservoir Environment, Chongqing Institute of Green and Intelligent Technology, Chinese Academy of Sciences.No. 266 Fangzheng Avenue, Shuitu Hi-tech Industrial Park, Beibei, Chongqing 400714, China.†

*Chlorella vulgaris*(FACHB-32) were used in the experiment. It was enriched by batch cultivation in an  Erlenmeyer flask with a volume of 250 mL, shaking 3~4 time every day manually. Algal samples were diluted to a cell density of 1×10^5^ cells/mL before seeding. Microwell arrays were pretreated with high-speed double-distilled water to expel air in the wells and were then placed in a glass petri dish with 50 mL of algal samples. Cells were sorted randomly in an independent fashion into individual microwells by gravity. After seeding, microwell arrays were carefully taken out with tweezers, washed carefully in sterile BG11 medium and then placed the PDMS microwell array chip in a confocal petri dish with 4 mL of sterile BG11 medium. Pictures were taken at 10× magnification on an inverted Olympus microscope (Olympus IX73, Japan). Statistics were made right after the rinsing step. Microwells with cells were counted, and statistical assessments were made on different microwell arrays.

Figure S1 and Figure S2 presented the cell distributions of *C. vulgaris* on various dimensions of microwell arrays. It can be clearly identified that cell distribution was quite different from that in the manuscript. This was possibly because algal cells from different cultivation procedures can have different sedimentation performances.
